# Macrophage CD40 plays a minor role in obesity-induced metabolic dysfunction

**DOI:** 10.1371/journal.pone.0202150

**Published:** 2018-08-10

**Authors:** Suzanne A. B. M. Aarts, Myrthe E. Reiche, Myrthe den Toom, Linda Beckers, Marion J. J. Gijbels, Norbert Gerdes, Menno P. J. de Winther, Esther Lutgens

**Affiliations:** 1 Amsterdam UMC, University of Amsterdam, Amsterdam Cardiovascular Sciences, Department of Medical Biochemistry, Amsterdam, The Netherlands; 2 Department of Biochemistry, University of Maastricht, Maastricht, The Netherlands; 3 Institute for Cardiovascular Prevention (IPEK), Ludwig Maximilian University (LMU), Munich, Germany; 4 Division of Cardiology, Pulmonology, and Vascular Medicine, Medical Faculty, University Hospital Düsseldorf, Düsseldorf, Germany; Technische Universitat Dresden, GERMANY

## Abstract

Obesity is a low-grade inflammatory disease that increases the risk for metabolic disorders. CD40-CD40L signaling plays a central role in obesity-induced inflammation. Genetic deficiency of CD40L in diet-induced obesity (DIO) ameliorates adipose tissue inflammation, hepatic steatosis and increases insulin sensitivity. Unexpectedly, absence of CD40 worsened insulin resistance and caused excessive adipose tissue inflammation and hepatosteatosis. To investigate whether deficiency of macrophage CD40 is responsible for the phenotype observed in the CD40^-/-^ mice, we generated CD40^flfl^LysM^cre^ and fed them a standard (SFD) and 54% high fat obesogenic diet (HFD) for 13 weeks. No differences in body weight, adipose tissue weight, adipocyte size, plasma cholesterol or triglyceride levels could be observed between CD40^flfl^LysM^cre^ and wild type (WT) mice. CD40^flfl^LysM^cre^ displayed no changes in glucose tolerance or insulin resistance, but had higher plasma adiponectin levels when fed a SFD. Liver weights, liver cholesterol and triglyceride levels, as well as the degree of hepatosteatosis were not affected by absence of macrophage CD40. CD40^flfl^LysM^cre^ mice displayed a minor increase in adipose tissue leukocyte infiltration on SFD and HFD, which did not result in differences in adipose tissue cytokine levels. We here show that loss of macrophage CD40 signaling does not affect obesity induced metabolic dysregulation and indicates that CD40-deficiency on other cell-types than the macrophage is responsible for the metabolic dysregulation, adipose tissue inflammation and hepatosteatosis that are observed in CD40^-/-^ mice.

## Introduction

Obesity is a worldwide epidemic and is associated with different metabolic diseases [1]. The low-grade chronic inflammation that occurs during obesity is held responsible for the co-morbidities including nonalcoholic fatty liver disease, type 2 diabetes mellitus, cardiovascular diseases and cancer [2, 3]. Immune cells in lean adipose tissue have an anti-inflammatory immune status and control tissue integrity and metabolism [4]. During obesity, immune cells obtain pro-inflammatory characteristics, and recruited macrophages are polarized to secrete pro-inflammatory cytokines including TNF and IL-6 [4]. Furthermore, the number of CD8^+^ effector T-cells secreting IFNγ is increased, while the number of regulatory T cells (Tregs) during obesity is reduced, thereby aggravating metabolic dysfunction [4].

An important co-stimulatory dyad that is crucial in regulating inflammatory processes is CD40L-CD40. CD40 is predominantly expressed by antigen-presenting cells, but also on phagocytes, endothelial cells and adipocytes [5, 6]. The ligand for CD40, CD40L, is expressed by T-cells, platelets and as a soluble form (sCD40L) [5]. CD40-CD40L interactions have been shown to be pivotal in the pathogenesis of chronic inflammatory diseases including multiple sclerosis, atherosclerosis, arthritis, lupus (SLE) and diabetes [7]. Also in diet induced obesity, genetic or antibody mediated disruption of CD40L signaling ameliorated adipose tissue inflammation and insulin resistance [8]. In contrast, genetic deficiency of CD40 resulted in a worsened metabolic phenotype with increased insulin resistance (IR), exacerbated adipose tissue inflammation and liver steatosis [9–12]. In particular, deficiency of CD40 resulted in increased macrophage accumulation in the adipose tissue [11], with a higher fraction of pro-inflammatory (M1) macrophages [9, 10] expressing increased levels of IL-6, IL-12, TNF and MCP-1 [9, 12]. Pro-inflammatory macrophages play an important role in adipose tissue inflammation during obesity [13, 14], as they overexpress genes important in macrophage migration and phagocytosis including TNF, iNOS and IL-6 [13, 14].

Ligation of macrophage CD40 is known to induce pro-inflammatory cytokine and chemokine secretion [15]. Moreover, CD40 ligation on macrophages upregulates expression of MHCII molecules and other co-stimulatory molecules, increases the production of inducible nitric oxide (iNOS) and enhances matrix metalloproteinase (MMP) expression [15]. The results observed in the CD40^-/-^ mouse DIO studies are in contrast with these known functions of macrophage CD40. In DIO, CD40 deficiency results in a pro-inflammatory phenotype in the adipose tissue and other metabolic organs [9]. To further explore the specific role of macrophage CD40 in adipose tissue inflammation we here subject CD40^flfl^LysM^cre^ and WT mice to diet-induced obesity.

## Material and methods

### Mice

Macrophage specific CD40 deficient mice (CD40^fl/fl^LysM^cre^) were generated by crossbreeding CD40^flfl^ mice (Ozgene Pty Ltd, Bentley, Australia) with LysM-cre mice [16]. CD40^flfl^ mice were custom created by inserting loxP sites upstream of exon 2 and downstream of exon 3. Littermates without the LysM-cre driver, CD40^flfl^LysM^wt^ (WT), were used as controls. All mice were bred and maintained at the animal facility of the Academic Medical Centre, Amsterdam (AMC).

### *In vitro* bone marrow derived macrophage culture

Bone marrow cells from CD40^flfl^LysM^cre^ and CD40^flfl^ mouse were harvested and cultured in RPMI-1640 with 2 mM L-glutamine, 10% fetal calf serum (FCS), penicillin (100 U/mL), streptomycin (100 μg/mL) (Gibco), and 15% L929-conditioned medium. Bone marrow derived macrophages (BMDMs) were harvested on day 8 and seeded at 1*10^6^ cells/mL. BMDMs were stimulated with FGK45 (CD40 agonistic antibody, 30μg/ml), IFNy (5ng/mL) and LPS (100ng/mL). After 6 hours cells were incubated with Fc blocking primary antibody CD16/CD32 (eBioscience) diluted in FACS buffer (0.5% bovine serum albumin (BSA), 2 mM EDTA, and 0.01% NaN_3_ in PBS). Cells were then incubated with fluorescently labelled CD40 antibody (BioLegend), and staining was analyzed by flow cytometry (FACSCanto II, BD Biosciences, Breda, The Netherlands) and FlowJo software version 7.6.5. (Tree star).

### Study design

Obesity was induced in the CD40^fl/fl^LysM^cre^ and WT mice by feeding a high fat diet (HFD; SNIFF-D12492 Energy 22% from carbohydrates, 24% from protein, and 54% from fat) for 13 weeks. Control mice were fed a standard fat diet (SFD; SNIFF-D12450B Energy 65% from carbohydrates, 26% from protein, and 9% from fat) during the same period. Mice had *ad libitum* access to food and water, and the condition and body weight of the mice were monitored weekly. Mice were anesthetized after 13 weeks of high fat or standard fat diet, by intraperitoneal injection of ketamine (100 mg/kg) and xylazine (16 mg/kg). Blood samples were taken for analysis by flow cytometry and for collection of plasma and mice were then intracardially perfused with PBS. Epidydimal adipose tissue (EpAT), subcutaneous adipose tissue (ScAT), liver, spleen, and lymph nodes were removed after sacrifice and used for subsequent analyses. All the experimental procedures were approved by the Ethical Committee for Animal Experiments of the Academic Medical Centre, Amsterdam (AMC).

### Glucose and insulin tolerance test

Glucose (GTT) and insulin tolerance tests (ITT) were performed after 12 weeks of dietary exposure. Mice were fasted for 6 hours for the GTT, and then injected i.p. with glucose (1mg/g body weight, Sigma-Aldrich). For the ITT 4 hour fasted mice were injected i.p. with insulin (1.1mU/g body weight, Sigma-Aldrich). Blood glucose levels were measured from whole blood using a glucometer (Bayercontour, Basel, Switzerland) at times indicated in Fig 2. Mouse fasting plasma insulin levels were measured in samples from 6 hour fasted mice by using an insulin ELISA kit (Mercodia, Uppsala, Sweden) following manufacturers’ protocol.

### Plasma adipokine and liver enzyme measurements

Adipokine levels in the plasma were measured using the mouse leptin ELISA Kit (ChrystalChem) and the mouse adiponectin ELISA Kit (AssayPro). Plasma levels of liver enzymes were measured using an alanine aminotransferase (ALT) ELISA Kit (Biomatik), and a mouse aspartate aminotransferase (AST) ELISA Kit (Biomatik).

### Cholesterol and triglyceride measurements

Plasma and liver total cholesterol and triglyceride (TG) concentrations were measured by standard enzymatic methods (CHOD-PAP and GPO-PAP; Roche Diagnostics). Frozen livers were homogenized in 1ml of SET buffer (250mM sucrose, 2mM EDTA, and 10mM Tris), and the samples then underwent multiple freeze-thaw cycles for cell-destruction.

### Flow cytometry

White blood cells from the stromal vascular fraction (SVF) from EpAT were analyzed by flow cytometry. Adipose tissue was minced into small pieces and digested with Liberase (0.25 mg/mL, Roche) for 45 minutes at 37°C. The digested samples were passed through a 70-μm nylon mesh (BD Biosciences, Breda, the Netherlands). The SVF was obtained from the resulting pellet and resuspended in FACS buffer. Erythrocytes in blood and spleen were removed by incubation with hypotonic lysis buffer (8.4 g of NH4Cl and 0.84 g of NaHCO3 per liter of distilled water). To prevent non-specific binding of antibodies to the Fc receptor, all cell suspensions were incubated with CD16/32 antibody (eBioscience) in FACS buffer. CD45 (eFluor450, and APC-Cy7, eBioscience), CD19 (PerCP-Cy5.5, eBioscience), CD3 (BV510, BioLegend), CD11b (PE-Cy7, BD Pharmingen), Ly6G (Percp-Cy5.5, BD Pharmingen), SiglecF (PE, BD Pharmingen), F4/80 (FITC, AbD serotec), and Ly6C (V450, eBioscience) antibodies were incubated with the SVF. Fluorescence was measured by flow cytometry (FACSCanto II, BD Biosciences, Breda, The Netherlands) and analyzed with FlowJo software version 7.6.5. (Tree star).

### Histology

EpAT and liver were collected, fixed in 4% paraformaldehyde and embedded in paraffin. Adipocyte size was measured on EpAT H&E stained sections and IHC on EpAT was performed for CD45 (BD), Mac3 (BD), and CD3 (AbD serotec). Additionally, livers were embedded in OCT and frozen at -80 degrees Celsius. To measure liver lipid content, 5 μm thick cryosections of the liver were stained with Oil red O (Sigma-Aldrich). Liver steatosis was graded on 4 μm thick haematoxylin-eosin (H&E) stained sections. Grades were scored 0–3 were 0 = no steatosis, 1 = mild steatosis, 2 = moderate steatosis, and 3 = severe steatosis. Analyses were performed by an observer who was blinded for the experimental conditions.

### Gene expression analysis

Total RNA of EpAT and liver was extracted using TRIzol (Invitrogen, Carlsbad, CA, USA. RNA was reverse transcribed with an iScript cDNA synthesis kit (Bio-Rad, Veenendaal, the Netherlands). qPCR was performed using a SYBR green PCR kit (Applied Biosystems, Leusden, the Netherlands) on a ViiA7 real-time PCR system (Applied Biosystems).

### Statistical analysis

7–16 mice per group were used for these experiments. Results are presented as means ± SEM. Data were analyzed by unpaired *t*-test, or Mann-Whitney U test where appropriate. Two-way ANOVA was used when comparing multiple groups using GraphPad Prism 7.0 software (GraphPad Software, Inc., La Jolla, CA, USA). P-values <0.05 were considered significant.

## Results

### Macrophage CD40 deficiency does not affect body weight gain during diet-induced obesity

**Fig 1:**
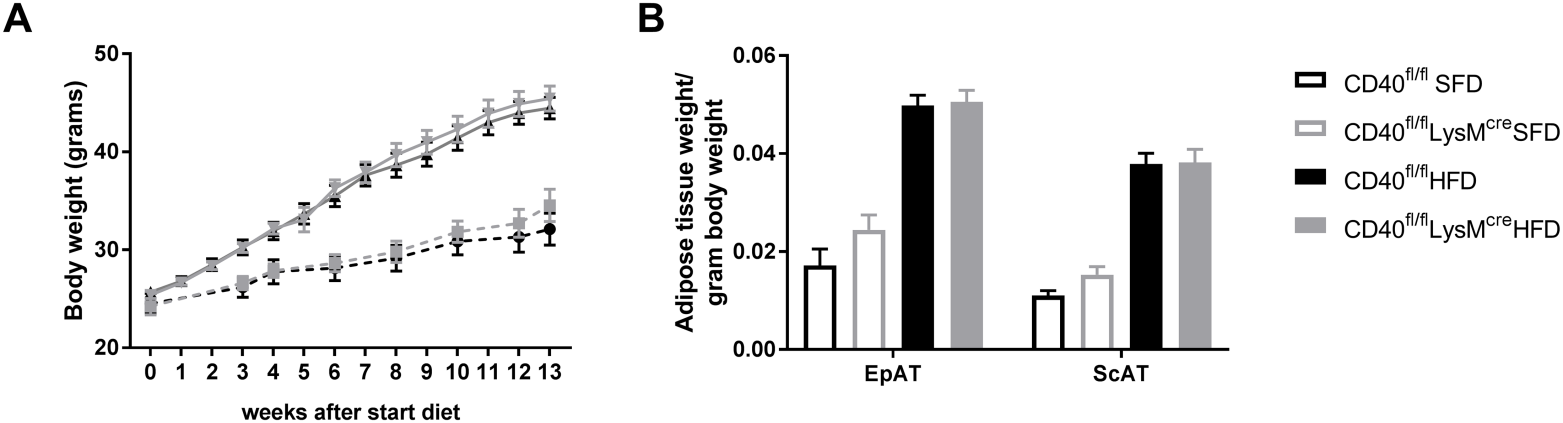
Body and adipose tissue weight of myeloid deficient mice. **(A)** Body weight of the high fat diet (HFD) CD40^fl/fl^LysM^cre^ and WT mice (n = 16/group) and the standard fat diet mice (SFD, n = 7/group) during 13 weeks of diet. **(B)** Tissue weights after 13 weeks of diet. Data is presented as mean ± SEM.

FACS analysis showed that CD40 expression on bone marrow derived macrophages (BMDMs) was sufficiently depleted from macrophages in the LysMcreCD40^fl/fl^ mice compared to wildtype mice, both under basal and activated conditions (78.1 ± 9.6% and 77.8 ± 4.4% reduction, respectively (S1 Fig). CD40^fl/fl^LysM^cre^ and WT mice were fed a 54% high fat diet (HFD) or a standard fat diet (SFD) for 13 weeks. There were no differences observed in weight gain between the groups on the same diet (Fig 1A). The weights of the epididymal (EpAT) and subcutaneous (ScAT) adipose tissues from the HFD groups had increased compared to the SFD groups, but they did not differ between the CD40^fl/fl^LysM^cre^ and WT mice (Fig 1B).

### Macrophage CD40 does not affect glucose tolerance or insulin resistance

**Fig 2:**
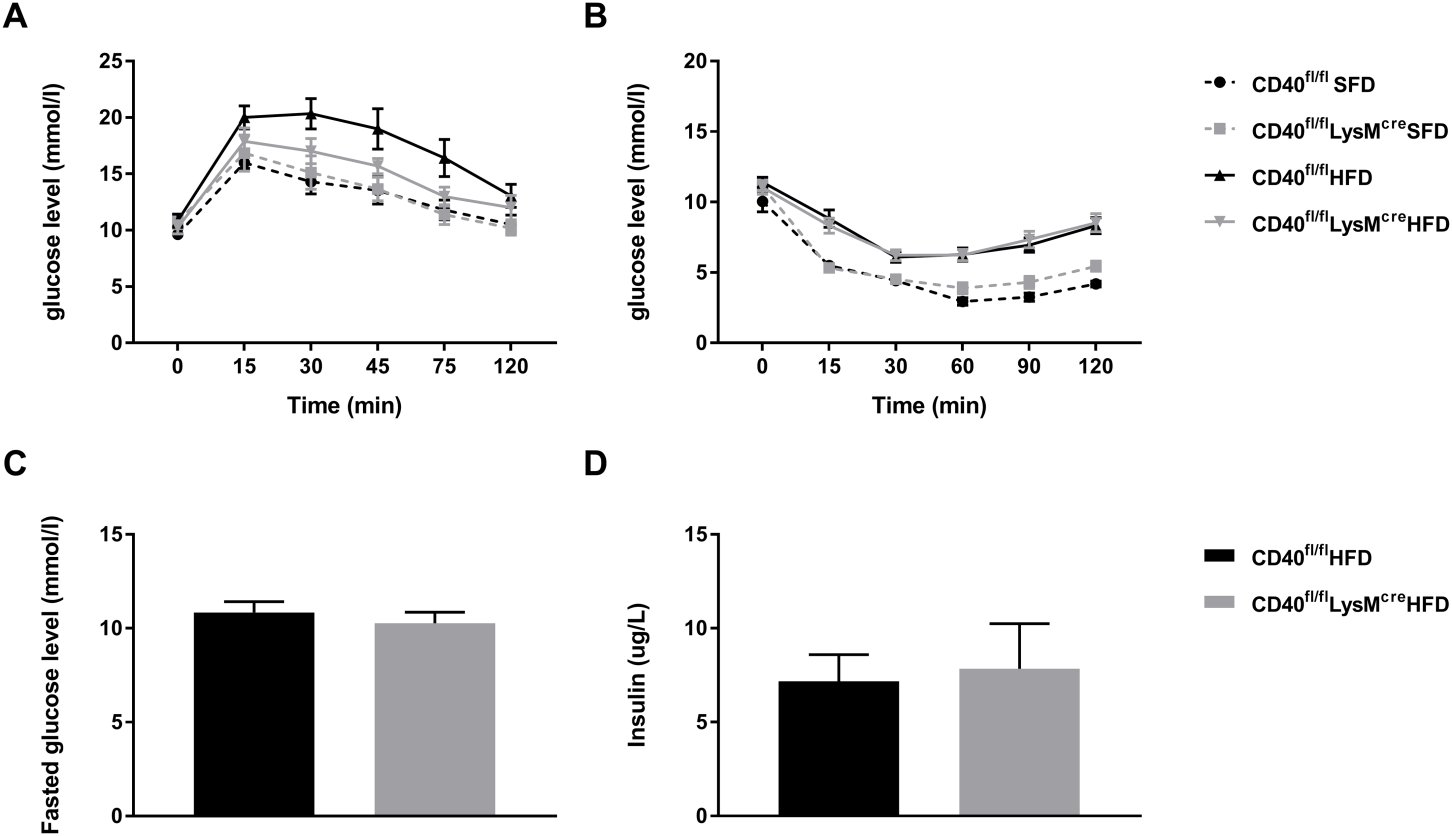
CD40^fl/fl^LysM^cre^ mice have similar glucose tolerance and insulin resistance than their WT controls. **(A)** Glucose tolerance test (n = 6–9 mice/group). **(B)** Insulin tolerance test (n = 6–16 mice/group). **(C)** Fasted glucose levels (n = 6–9 mice/group). **(D)** Fasted insulin levels (n = 5–8 mice/group). Data is presented as mean ± SEM.

Glucose and insulin tolerance tests were performed after 12 weeks of dietary intervention. As expected, 54% HFD feeding worsened glucose sensitivity and insulin resistance compared to SFD feeding (Fig 2A and 2B). HFD fed CD40^fl/fl^LysM^cre^ mice had a slightly better glucose sensitivity compared to their WT controls on a HFD, although this difference was not significant (area under the curve WT mice = 87.7 vs 74.7 for CD40^fl/fl^LysM^cre^ mice p = 0.142, Fig 2A). No differences in insulin resistance could be observed between CD40^fl/fl^LysM^cre^ and WT mice (Fig 2B). Fasted glucose and insulin levels did not differ between the HFD groups (Fig 2C and 2D).

### Adipose tissue function was not affected by loss of macrophage CD40 function

**Fig 3:**
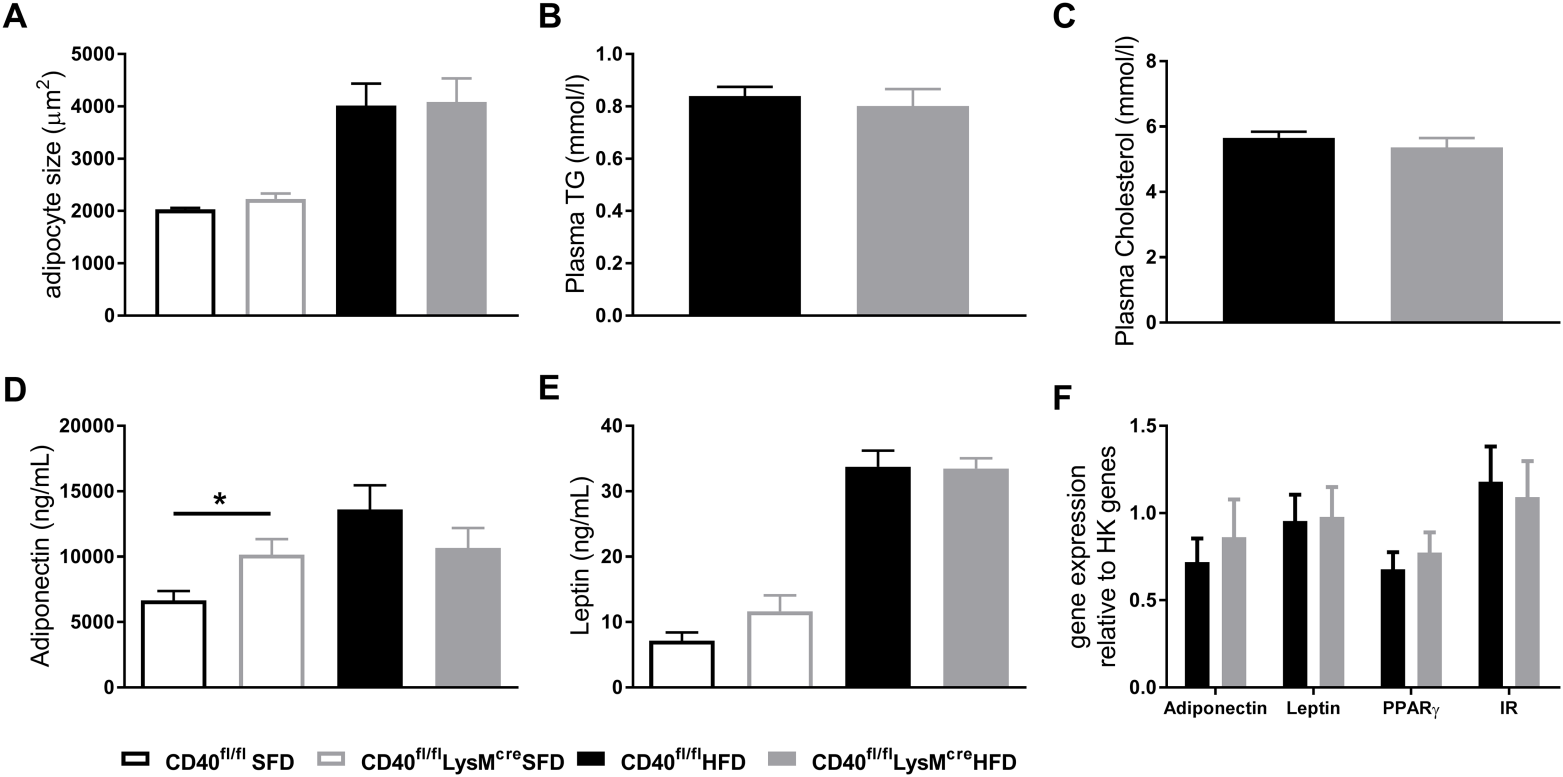
Adipose tissue function is not affected by macrophage CD40 deficiency. **(A)** Adipocyte size after 13 weeks of dietary intervention. **(B)** Plasma triglycerides (TG). **(C)** Plasma total cholesterol levels. **(D)** plasma adiponectin levels are increased in lean macrophage CD40 deficient mice but are not different in obese mice. **(E)** Plasma leptin levels. **(F)** mRNA expression of adiponectin, leptin, PPARγ and insulin receptor (IR) in EpAT, (n = 6–9 mice/group). Data is presented as mean ± SEM. *p<0.05, as determined by non-parametric Mann Whitney test.

HFD feeding did increase adipocyte size, but no differences were found between CD40^fl/fl^LysM^cre^ and WT mice fed a similar diet (Fig 3A). No differences were found for plasma TG and total cholesterol (Fig 3B and 3C). Plasma adiponectin levels were elevated in CD40^fl/fl^LysM^cre^ when fed a SFD, but this difference disappeared after HFD intervention (Fig 3D). Plasma leptin levels were increased in both groups upon HFD feeding, but macrophage CD40 had no effect on leptin levels (Fig 3E). Analysis of EpAT show no difference in gene expression levels of adiponectin, leptin, PPARγ and the insulin receptor (IR) during HFD (Fig 3F).

### Adipose tissue inflammation is slightly increased in CD40^flfl^LysM^cre^ mice

**Fig 4:**
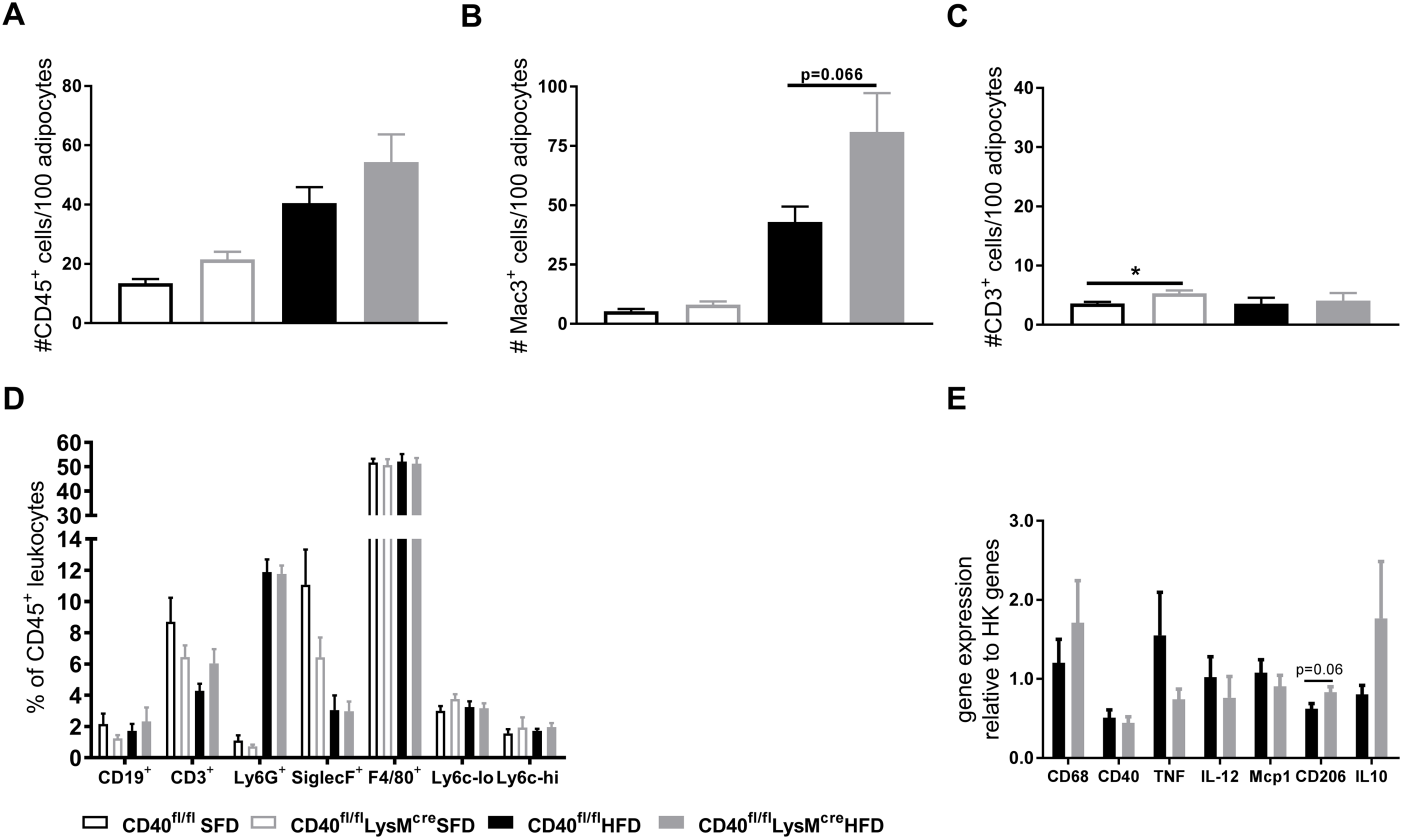
Adipose tissue inflammation is slightly increased in macrophage CD40 deficient mice without resulting in altered cytokine release. **(A)** Quantification of CD45 staining for leukocytes of EpAT (n = 16 for HFD and n = 7 for SFD). **(B)** Quantification of Mac3 staining for macrophages in EpAT (n = 16 for HFD and n = 7 for SFD). **(C)** Quantification of CD3^+^ staining for T cells in EpAT (n = 16 for HFD and n = 7 for SFD). **(D)** Flow cytometric analysis of leukocyte subsets in EpAT, as % of total CD45^+^ leukocytes (n = 7-9/group). **(E)** mRNA expression of inflammatory markers in EpAT (n = 7-9/group). Data is presented as mean ± SEM.

Histological analysis showed that the deficiency of macrophage CD40 did not affect the amount of CD45^+^ leukocytes that infiltrated epAT in lean or obese mice (Fig 4A and S2 Fig). However, epAT of HFD fed CD40^flfl^lysM^cre^ mice contained slightly more mac3^+^ macrophages, and epAT of lean CD40^flfl^lysM^cre^ mice contained a little more CD3^+^ T cells (Fig 4B and 4C and S2 Fig). Flow cytometric analysis of the stromal vascular fraction (SVF) showed that there were no significant differences in leukocyte subsets between the groups (Fig 4D). In line with this, transcripts of inflammatory markers in the adipose tissue showed no significant changes. However, epAT of CD40^fl/fl^LysM^cre^ mice contained slightly lower TNF, IL-12 and Mcp1, and slightly elevated levels of CD206 and IL-10 mRNA (Fig 4E).

### Loss of macrophage CD40 expression does not affect development of hepatosteatosis during DIO

**Fig 5:**
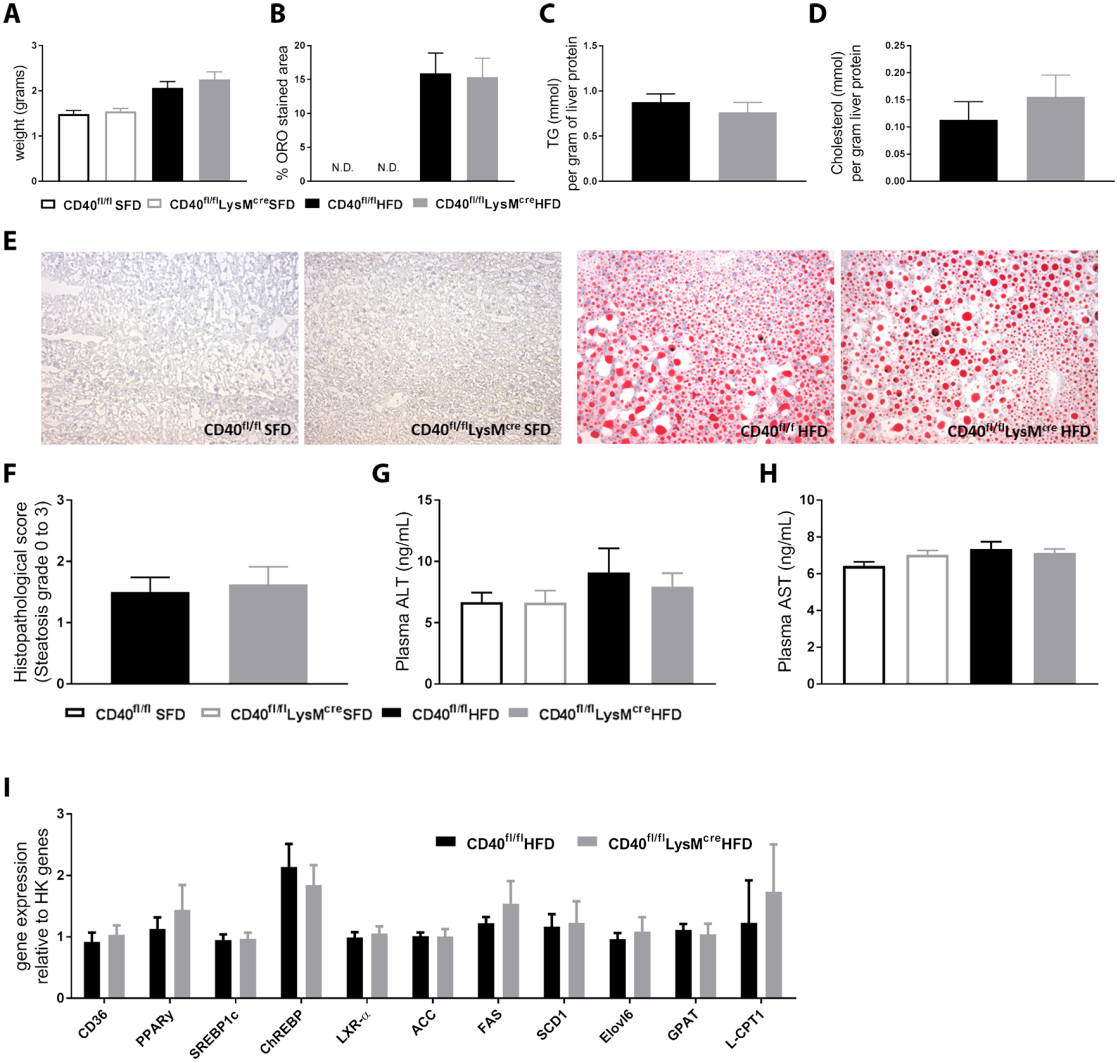
Lipid accumulation was not affected by deficiency of macrophage CD40. **(A)** Liver weight (n = 16 for HFD and n = 7 for SFD) **(B)** Quantification of oil-red-o stained area in the liver (n = 16 for HFD and n = 7 for SFD). **(C)** Liver TG levels (n = 6-9/group). **(D)** Liver total cholesterol levels (n = 6-9/group). **(E)** Representative pictures of ORO staining of the livers. **(F)** Histopathological score of liver steatosis (n = 16/group). **(G)**. Plasma ALT levels (n = 7-9/group). **(H)**. Plasma AST levels (n = 7-9/group). **(I)** mRNA expression of liver lipid metabolism genes (n = 7-10/group). Data is presented as mean ± SEM. N.D. means ‘not detectable’.

Liver weights were increased upon HFD feeding but did not differ between the groups (Fig 5A). Lipid accumulation measured by quantification of oil-red-o (ORO) stained area of the liver was increased during diet-induced obesity but did not differ between the HFD groups (Fig 5B). In line with these results, liver TG (Fig 5C) and liver total cholesterol (Fig 5D) was not different in CD40^fl/fl^LysM^cre^ mice compared to WT mice. Representative pictures of lipid accumulation in the liver are shown in Fig 5E. The degree of hepatosteatosis was the same for both groups on the HFD (Fig 5F), and plasma levels of liver enzymes alanine aminotransferase (ALT, Fig 5G) and aspartate aminotransferase (AST, Fig 5H) show no differences in liver damage between the groups. mRNA expression of genes involved in liver lipid metabolism were not different between WT and CD40^fl/fl^LysM^cre^ mice on HFD (Fig 5I).

## Discussion

Pro-inflammatory macrophages play an important role in adipose tissue inflammation during obesity and its associated metabolic diseases [13, 14]. High fat diet induced recruited adipose tissue macrophages overexpress inflammatory genes including TNF, iNOS and IL-6, and have increased lipid content [13, 14]. While genetic ablation of CD40L ameliorates the metabolic syndrome, CD40^-/-^ mice have an unexpected worsened obese phenotype [9]. Upon high fat diet feeding, CD40 deficient mice displayed increased macrophage accumulation in the adipose tissue, with a higher fraction of pro-inflammatory (M1) macrophages, and exhibited metabolic dysregulation [9]. Here, we investigated the role of macrophage CD40 in diet-induced obesity using CD40^flfl^LysM^cre^ mice. Surprisingly, we found that CD40^flfl^LysM^cre^ mice on a high fat diet showed the same phenotype as their WT counterparts.

The minor phenotype of the macrophage CD40 deficient mice in diet-induced obesity might be caused by the differential involvement of TRAF-adaptor molecules essential in CD40 signaling [9]. TRAF-1 expression is upregulated during adipose tissue inflammation, and TRAF-1–deficient mice were protected from DIO and its metabolic consequences [17]. Mice with deficient hepatic TRAF-2 expression have decreased blood glucose levels and impaired glucagon signaling under high fat diet conditions, while TRAF-2 overexpression enhanced the hyperglycemic response to glucagon [18], indicating an important role for hepatic TRAF-2 in hepatic gluconeogenesis. Deficiency of CD40-TRAF2/3/5 signaling resembled the phenotype of CD40 deficiency in DIO, and worsened diet-induced obesity. In contrary, deficiency of CD40-TRAF6 signaling ameliorated IR, liver steatosis, and inflammation of adipose tissue related to obesity and thus resembled the phenotype of CD40L deficiency [9]. Pharmacologic inhibition of the CD40-TRAF6 pathway using the small molecule inhibitor (SMI) 687002 ameliorated obesity-related metabolic complications and type 2 diabetes [9, 19, 20]. The CD40-TRAF6 pathway is mostly important in macrophages [21–23], therefore we hypothesized that the CD40^flfl^LysM^cre^ mice would show the same phenotype as the CD40-TRAF6 deficient mice.

We now learned that only when macrophage CD40-TRAF6 signaling is deficient, DIO is ameliorated [9], and deficiency of the whole CD40 signaling in macrophages does not affect DIO related abnormalities. In the CD40^flfl^LysM^cre^ mice both the CD40-TRAF6 and the CD40-TRAF2/3/5 signaling pathways are blocked in the macrophages. As blockade of CD40-TRAF2/3/5 signaling in diet-induced obesity worsened metabolic complications [9], it could be that the beneficial effects of CD40-TRAF6 blockade are counteracted by the lack of CD40-TRAF2/3/5 signaling.

Explanations for the exacerbated adipose tissue inflammation in the CD40^-/-^ mice are sought amongst others, in the increased presence of endothelial markers CD31 and von Willebrand factor in the adipose tissue of CD40-/- mice, increasing the leukocyte entry sites into the adipose tissue [10]. Other studies suggest that not endothelial cells, but leukocyte CD40 causes the pro-inflammatory phenotype and confirmed this theory by feeding a HFD to CD40 BM chimeric mice [12]. Moreover, increased numbers of T cells were observed in obese CD40^-/-^ mice [10, 11], leading to studies were B and T cell deficient RAG1-/- mice are transferred with CD40 deficient bone marrow [11, 12]. These studies shown that CD40 deficiency on immune cells aggravated obesity and insulin resistance [11, 12]. Furthermore, a specific role for CD8+ T cells was found [12]. RAG^-/-^ mice transferred with CD40-deficient CD8+ T cells exhibited worsened glucose tolerance and insulin sensitivity compared with mice receiving WT CD8+ T cells [12].

In line with the phenotype of the CD40^flfl^LysM^cre^ mice, hematopoietic disruption of CD40 did decrease adipose tissue T-cell accumulation but did not affect obesity-induced insulin resistance in mice [24], indicating that non-hematopoietic cell CD40 expression probably contributes to the phenotype observed in the total body CD40 knock out mice. Moreover, we should keep in mind that the CD40^flfl^LysM^cre^ mice have only 80% reduction of CD40 expression on macrophages, and it might be possible that the remaining 20% could exert sufficient signaling to induce the effects that were blocked in the other models.

In conclusion, we here show that loss of macrophage CD40 signaling does not significantly affect diet-induced obesity.

## Supporting information

S1 Fig CD40 expression was decreased on macrophages from CD40^flfl^LysM^cre^ compared to WT mice. CD40 mean fluorescence intensity (MFI) was measured by flow cytometry after bone marrow derive macrophages were stimulated with CD40-stimulating antibody FGK45 and IFNγ and LPS (n = 5/group). Data is presented as mean ± SEM. *p<0.05, **p<0.01 as determined by non-parametric Mann Whitney test.
(TIF)

S2 Fig Representative pictures of CD45, Mac3, and CD3 staining of epAT from CD40^flfl^LysM^cre^ and WT mice. Scale bar is 100 μm.
(TIF)

S1 Checklist ARRIVE guidelines checklist animal research. (PDF)
