# Supplementary figures and images for: Macrophage CD40 plays a minor role in obesity-induced metabolic dysfunction
Source: PLoS One. 2018 Aug 10;13(8):e0202150. doi: 10.1371/journal.pone.0202150 (PMC6086432; doi:10.1371/journal.pone.0202150)

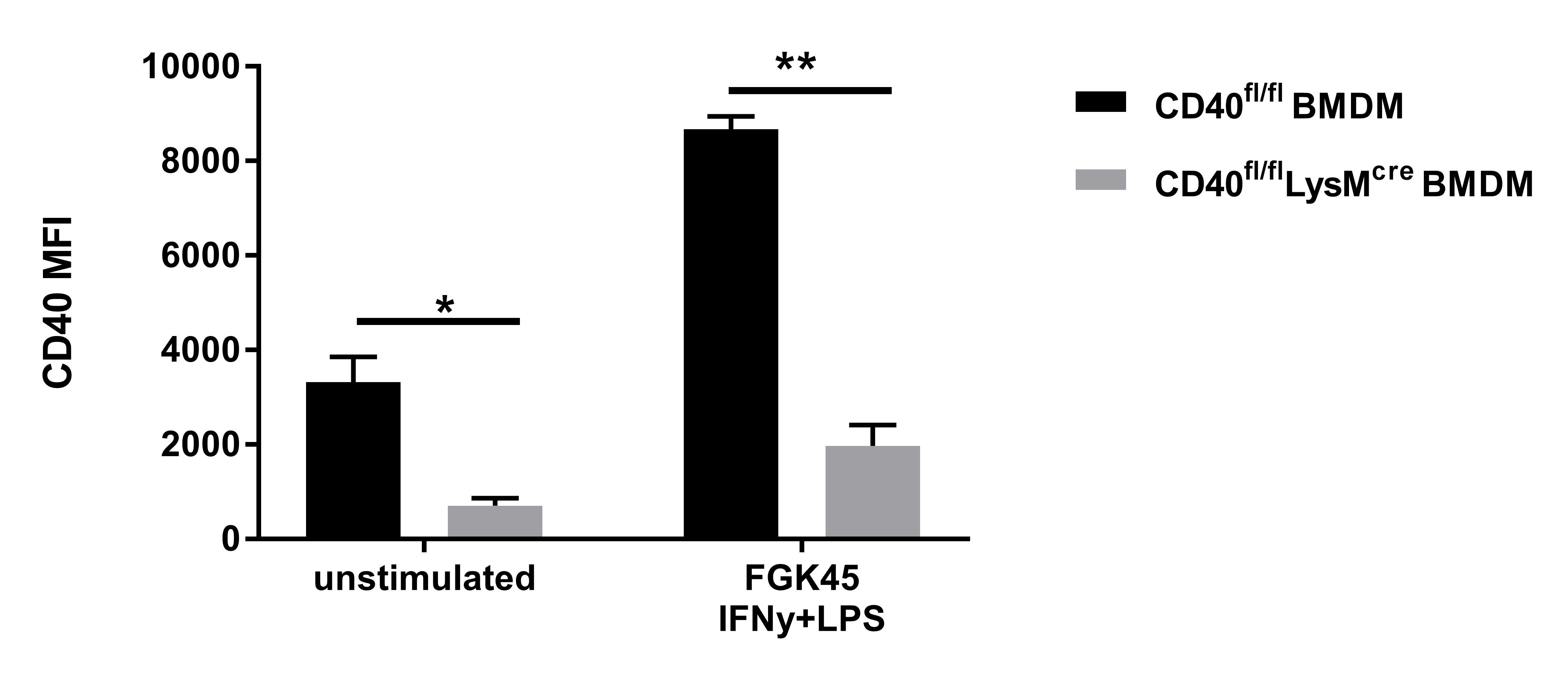

Supplement: S1 Fig — CD40 mean fluorescence intensity (MFI) was measured by flow cytometry after bone marrow derive macrophages were stimulated with CD40-stimulating antibody FGK45 and IFNγ and LPS (n = 5/group). Data is presented as mean ± SEM. *p<0.05, **p<0.01 as determined by non-parametric Mann Whitney test. (TIF) [file pone.0202150.s001.tif]

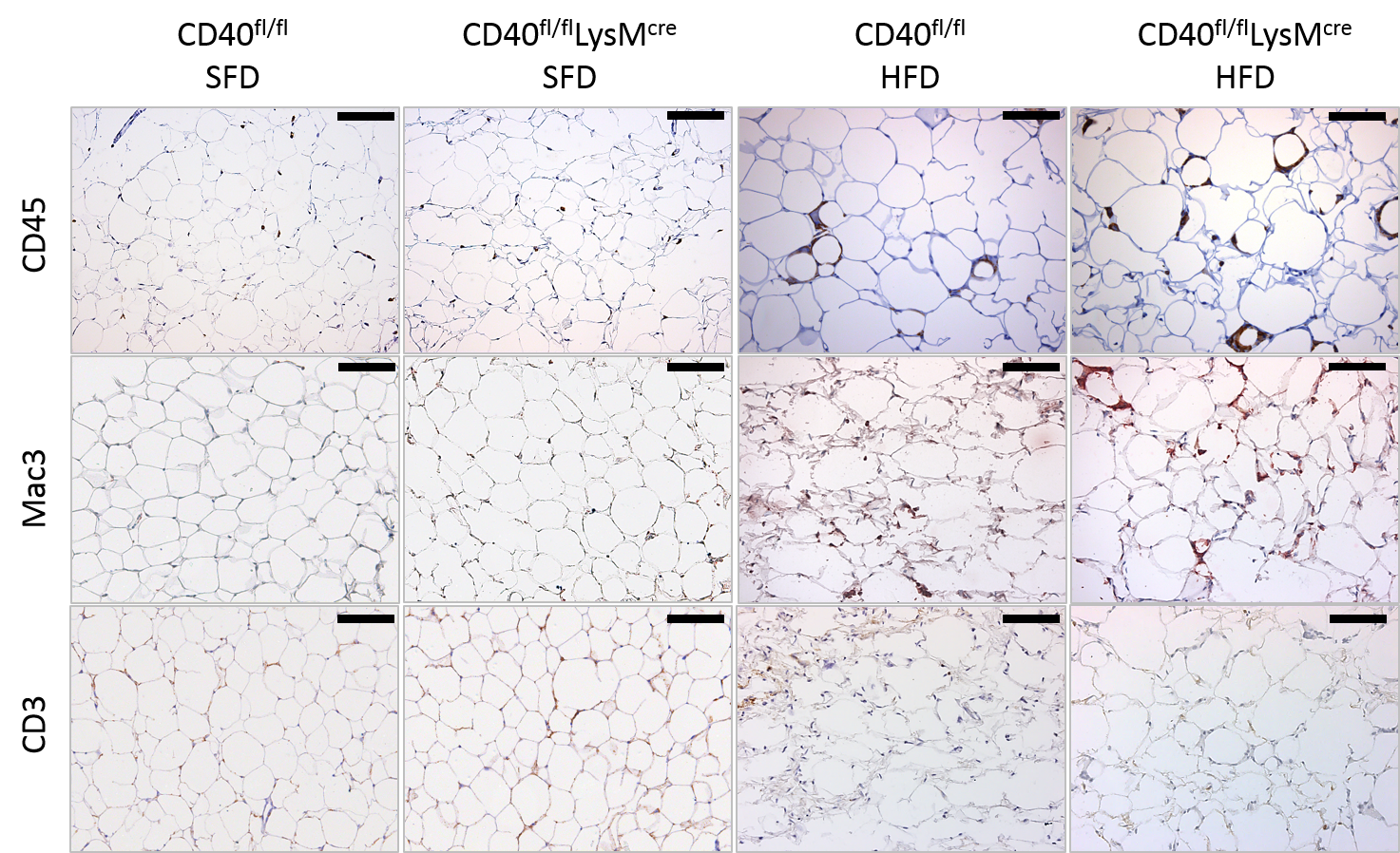

Supplement: S2 Fig — Scale bar is 100 μm. (TIF) [file pone.0202150.s002.tif]
